# Driving Behaviour Analysis Using Machine and Deep Learning Methods for Continuous Streams of Vehicular Data

**DOI:** 10.3390/s21144704

**Published:** 2021-07-09

**Authors:** Nikolaos Peppes, Theodoros Alexakis, Evgenia Adamopoulou, Konstantinos Demestichas

**Affiliations:** Institute of Communication and Computer Systems, Zografou, 15773 Athens, Greece; npeppes@cn.ntua.gr (N.P.); talexakis@cn.ntua.gr (T.A.); eadam@cn.ntua.gr (E.A.)

**Keywords:** driving behaviour analysis (DBA), machine learning (ML), deep learning (DL), data streaming, vehicle sensors

## Abstract

In the last few decades, vehicles are equipped with a plethora of sensors which can provide useful measurements and diagnostics for both the vehicle’s condition as well as the driver’s behaviour. Furthermore, the rapid increase for transportation needs of people and goods together with the evolution of Information and Communication Technologies (ICT) push the transportation domain towards a new more intelligent and efficient era. The reduction of CO_2_ emissions and the minimization of the environmental footprint is, undeniably, of utmost importance for the protection of the environment. In this light, it is widely acceptable that the driving behaviour is directly associated with the vehicle’s fuel consumption and gas emissions. Thus, given the fact that, nowadays, vehicles are equipped with sensors that can collect a variety of data, such as speed, acceleration, fuel consumption, direction, etc. is more feasible than ever to put forward solutions which aim not only to monitor but also improve the drivers’ behaviour from an environmental point of view. The approach presented in this paper describes a holistic integrated platform which combines well-known machine and deep learning algorithms together with open-source-based tools in order to gather, store, process, analyze and correlate different data flows originating from vehicles. Particularly, data streamed from different vehicles are processed and analyzed with the utilization of clustering techniques in order to classify the driver’s behaviour as eco-friendly or not, followed by a comparative analysis of supervised machine and deep learning algorithms in the given labelled dataset.

## 1. Introduction

The rapid growth of transportation needs, for both people and goods, as well as the evolution of Information and Communication Technology (ICT) has led to new standards concerning the transportation domain. Moreover, the evolution and the maturity of sensor technologies enable the establishment of new transportation paradigms which can offer better understanding of driving behaviour. Adding to that, the growing number of vehicles moving daily especially in the urban centers poses new threats for both people and the environment. For example, in the European Union countries around 242.7 million passenger cars were reported by the European Automobile Manufacturers Association in 2019 [1]. It is worth mentioning that the World Health Organization (WHO) in 2020 reported that in 2016 about 1.35 million people lost their lives in car crashes worldwide [2]. Adding to the increasing number of vehicles and car accidents, the environmental footprint of vehicles should be also examined. More specifically, as reported by European Parliament in 2019, transport is responsible for nearly the 30% of the European Union’s total carbon dioxide (CO_2_) emissions [3]. Taking all the aforementioned facts into account, it becomes clear that research for safer and environmental-friendly practices and policies should intensify so as to improve emerging environmental and safety threats. In this direction, monitoring and analysis of vehicular data originating from in-vehicle sensors can provide useful insights and predict possible menacing driving behaviour both in environmental and safety terms.

During the last decades, governments around the world commenced actions so as to reduce CO_2_ emissions and slow down, if not prevent, climate change and its disastrous consequences. More specifically, the European Union (EU) adopted various measures so to achieve climate neutrality by 2050. One of these measures aims the reduction of car emissions by making the legislation tougher regarding emissions standards and by motivating Europeans to shift to hybrid and electric vehicles [4]. Besides the legislation and the shift to electric cars, which are measures mainly focused on future vehicle purchases, focus should also be given on the millions of internal combustion engine vehicles that are currently running on streets. Since driving behaviour is directly related to the emissions produced by vehicles, it is of utmost importance to gather and analyze data coming directly from the vehicle so as to assess and inform the driver about how his/her behaviour affects fuel consumption. This would provide an extra motive and a better understanding to the driver regarding driving in an eco-friendlier way.

The data needed for driving behaviour analysis are collected through on-board vehicle sensors. All vehicles manufactured during the last decades have various sensors installed in order to measure and monitor their speed, temperature, the engine’s revolutions per minute (rpm) and more. Until some years ago, these measurements would or could not be transferred outside the vehicle and their usage was only limited for in-car diagnostics purposes. The rapid development of ICT made it possible to enhance vehicles with networking capabilities so that they are now enabled to communicate with external devices such as smartphones, databases, other vehicles (vehicle-to-vehicle communication), emergency call centers, highways and more. Even the older vehicle models can transfer data to external applications by engaging on-board diagnostics (OBD) units. Especially, nowadays, the OBD II protocol is commonly used for diagnostics reading. Thus, even older vehicles can be equipped with a compact device which can gather different measurements and diagnostic messages and subsequently transfer them to a smartphone (e.g., via Bluetooth) or another device with networking capabilities in order to be stored and further analyzed to an external platform or database. Furthermore, alongside the rapid development of sensors and networking technologies there is a remarkable evolution in data streaming, storage and analysis technologies. The big data and artificial intelligence applications which now are commercially available have enabled real-time analysis of fast paced generated data such as those originating from vehicles.

In this light, this paper proposes and presents a novel approach for continuous data gathering, storage and analysis based on state-of-the-art streaming processes as well as big data and machine and deep learning technologies. The data used for the present study have been acquired from five different vehicles which belong to a highway administrator in Greece, thus cover long distances by different drivers each day, all of them equipped with OBD II units so as to collect and transmit the data. This data analysis, aiming at classifying drivers’ behaviour as eco-friendly or not, is performed in a cloud-based platform which is developed and deployed in order to facilitate the needs of the rapid and vast data streams coming from vehicles through a suitable streaming module. The analysis engages both well-known unsupervised machine learning (clustering) techniques for data labelling purposes as well as supervised machine and deep learning algorithms benchmarking in the pre-labelled dataset.

The remainder of this paper is structured as follows: Section 2 features a state-of-the-art literature review in the domain of driving behaviour analysis; Section 3 presents the equipment utilized as well as the architecture of the streaming processes and the cloud-based platform; Section 4 demonstrates the dataset and the algorithms employed for evaluation purposes on the given dataset while Section 5 presents the evaluation results and Section 6 concludes the paper and points to future work and extensions of the current study.

## 2. Related Works

**Figure 1:**
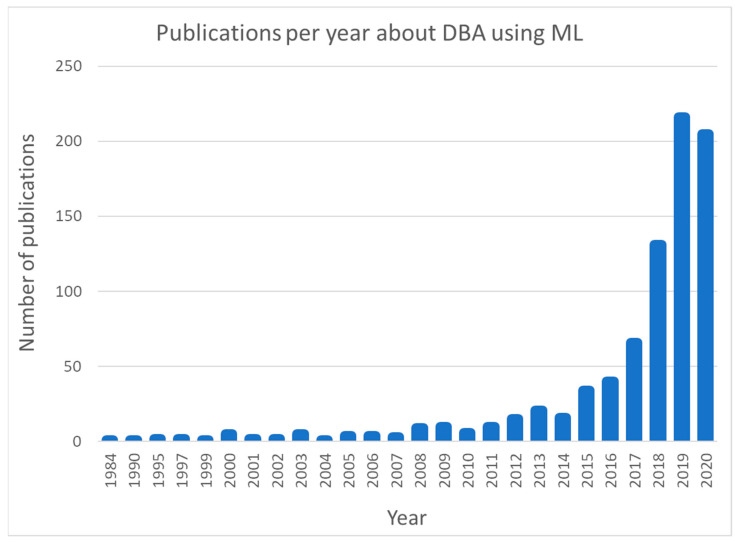
Publications per year according to SCOPUS searching term “TITLE-ABS-KEY (((driving AND behavior) OR (driving AND behaviour)) AND (machine AND learning))”.

In the last few years, driving behaviour analysis (DBA) through machine learning (ML) algorithms is an upward trend. More specifically, during the last decade this research topic shows a rapid growth as can be seen in Figure 1 which depicts the number of relevant publications as retrieved from SCOPUS by searching the term “TITLE-ABS-KEY (((driving AND behavior) OR (driving AND behaviour)) AND (machine AND learning))”. This upward trend is anticipated considering the rapid evolution of computing technology and the commercialization of machine learning methods. Also, the digitization of vehicles and the integration of ICT on them created a breeding ground for driver behaviour analysis and vehicle automation.

Driving behaviour analysis is a very complex problem that depends on various and divergent parameters such as road conditions, traffic, the vehicle itself, the driver, etc. Thus, approaches for addressing this problem feature numerous variations in its expression as well as the method followed to tackle it. Alluhaibi et al. [5] made an extensive literature review about different parameters and methods that can be used for DBA. More specifically, they focused their study on research efforts which classify the driver behaviour as aggressive (dangerous) or not (normal). The sensors employed for driver behaviour classification presented in [5] include: (i) in-vehicle data recording systems which refer mainly to systems engaging CAN-bus and OBD interfaces as well as other devices or equipment installed in the vehicle; (ii) smartphone-based sensing in vehicles, which relies mainly on the utilization of the plethora of sensors installed on a smartphone in order to assess driving behaviour; and last but not least the (iii) behaviour detection methods which is a wider category that can entail the other two and also focuses on real-time assessment methods and techniques by engaging statistical and machine learning algorithms. The authors of [5] concluded that the DBA can be classified as real-time or not and the outcome differs between methods based on the available resources and the distinct needs of each case.

In the last few years as shown in Figure 1 the engagement of machine learning is constantly gaining ground. Thus, in this direction Elassad et al. [6] performed a systematic literature review of relevant studies, which focus on proposing solutions and methods about DBA using machine learning. The authors of [6] categorized the ML techniques used in the following groups (in the parenthesis the percentage of studies containing each method is provided): (i) neural networks (NN) (22%); (ii) support vector machines (SVM) (24%); (iii) fuzzy & neuro fuzzy-based (3%); (iv) clustering (8%); (v) inductive rule-based (1%); (vi) instance-based (7%); (vii) decision trees (DT) (6%); (viii) Bayesian learners (15%); (ix) ensemble learners (11%); (x) evolutionary algorithms (1%) and (xi) miscellaneous (2%) based on 82 studies published in 46 different journals and conferences. Furthermore, in [6] detailed analyses concerning the metrics used in the studies such as accuracy, recall, precision, etc. as well as classification of the studies in three main categories based on DBA dimension (driving events, physiological state and psychological state) is provided. Elassad et al. [6] through their systematic literature review underpinned the emerging trend of ML methods applied for DBA as well as the gaps and the future directions in this research domain. Moreover, this research concluded that no unique solution that outperforms the others exists and that the performance of each method depends highly on the dataset and the context of the study.

The scenario for DBA concerning the ecological footprint is an open research issue due to the fact that despite the rapid evolution of ICT integration in vehicles, fuel consumption is highly dependent on the driver’s behaviour. In this direction, Araujo et al. [7] proposed a framework which combines measurements coming directly from the vehicle’s electronic control unit (ECU) sent via OBD II to a smartphone for analysis. The identified vehicle condition is combined with the user inputs regarding fuel consumption and through a fuzzy evaluator the android application presents to the user useful hints about his/her behaviour in order to further improve it [7]. Massoud et al. [8] proposed a solution which also utilizes an OBD II adapter to retrieve the data from car sensors and a smartphone so as to assist the driver with driving more efficiently in terms of fuel consumption. More specifically, authors of [8] took into account several parameters (including throttle position, RPM, speed and car jerk) in order to calculate a score from 0 to 100 (a high score indicates environmental-friendly driving). Drivers in this proposed framework receive real-time feedback when driving in a non-eco-friendly way. Last but not least, Massoud et al. [8] promote the idea of involving gamification of this process by ranking drivers for example, so as to give them an extra incentive to drive in a more fuel efficient way. A more detailed presentation of the aforementioned approaches, was also performed by Massoud [9] in her doctoral dissertation in which she provided more technical details of algorithms and technologies used. Chen et al. [10] focus on eco-driving behaviour analysis by engaging machine learning methods such as Principal Component Analysis (PCA) and multiple linear regression. In [10], authors considered the frequency of each driving event in a certain distance as an independent variable and vehicle fuel consumption as the dependent variable in the context of the aforementioned methods with the view to evaluate the driver behaviour and to provide useful eco-driving advices in order to improve their skills. It is worth mentioning that the environmental-friendly driving is an evolving research area and alongside the aforementioned studies there are also other frameworks which aim to study different aspects of it. In this light, the enviroCar is a crowdsourcing project which aims to create an open repository for vehicular data that can be used for further research by anyone interested in this topic [11]. Also, Delhomme et al. [12] followed a statistical approach based on data of 1243 French drivers, both men and women, from different age groups. This study revealed that age, gender, and environmental concern are found to influence the self-reported frequency of actions, and the perceived difficulty of anticipation, driving at a steady speed, conservative use of the accelerator, and gear shifting [12].

A similar approach using OBD II and smartphone was also performed by Castignani et al. in [13,14] but their proposal consists of a more generic DBA and does not focus solely on ecological terms. In [13] Castignani et al. proposed an Android-based solution which utilizes the data from android sensors in order to classify drivers based on twelve input variables, eighteen inference rules and an output fuzzy evaluator which considers three driving styles: normal, moderate and aggressive. A further step from the same authors was the SenseFleet application featured in [14]. SenseFleet is a framework which can detect different events (acceleration, braking, steering and over-speeding) by utilizing the data from GPS and OBD II. Moreover, the authors of [14] underpin that SenseFleet introduces a scoring algorithm which alongside the number of events takes into account context information such weather, road, traffic conditions etc. Abdelrahman et al. conducted a series of studies [15,16,17] which examine the performance of different machine learning algorithms on data gathered from an OBD II adapter, sent to a smartphone and then to a cloud-based environment for further analysis. These studies used the SHRP 2 dataset which is one of the largest datasets of driving data. Among these studies, random forests (RF)-based algorithms [16,17] as well as support vector regression (SVR) ones [15,16] featured better performance compared to other algorithms. Another interesting comparative study between different machine learning algorithms on vehicular data was performed by Chen et al. [18]. In this study, authors studied real data coming from an OBD II connector installed on a vehicle deployed for the needs of this research. The results of the different examined algorithms indicated that for this specific dataset the RF approach performed in the best way [18]. Also, a similar approach was performed by Navneeth et al. [19] who, through an OBD II connector and a smartphone, collected data and analyzed them using ML and especially the K-nearest neighbors (KNN) algorithm in order to cluster the driver behaviour and calculate a score which indicates how risky the driver’s behaviour is. Carvalho et al. [20] utilized only data that can be collected via the smartphone’s sensors such as acceleration, position, etc. in order to study the performance of different types of recurrent neural networks (RNNs). More specifically, they collected data from four different car routes performed by two different drivers under the same weather conditions. The evaluation of the RNNs was based on seven different driving events (labels) and indicated that the best results were achieved by the gated recurrent unit (GRU) over long short-term memory (LSTM) and simpleRNN for this specific dataset [20]. Obuhuma et al. studied DBA by using dynamic Bayesian networks on data collected by an on-board unit (OBU) which can be categorized in three categories i.e., acceleration, braking and cornering which affect the scoring process and the analysis of the driver’s behaviour [21]. Moreover, a very interesting study was conducted by Lindow and Kashevnik [22] who made a detailed literature review on existing studies and approaches about different sensors, technologies, data and machine learning methods used to study driver behaviour. In addition to this literature review, they propose a conceptual framework which engages smartphones to gather data, and a cloud infrastructure for data hosting and analysis through ML algorithms with the outputs of this analysis becoming available to different categories of end-users such as fleet managers, drivers, operational centers etc. [22].

In the previous paragraphs, several related interesting studies were presented. Firstly, the studies discussed were focused mainly on the ecological aspect of the driver behaviour while the next paragraph introduced studies which examined the DBA problem from a wider point of view also engaging machine learning methods. In addition to the aforementioned studies, there are some honorable mentions which supported the motivation, conceptualization as well as the implementation of the present study. Specifically, Khandakar et al. [23] presented a portable solution which engages OBD II connector with a smartphone to gather data in order to analyze them and track possible anomalies and distractions in driving behaviour. A very similar implementation approach was made by Renininger et al. [24] who engaged also an OBD II connector and a smartphone in order to collect vehicular data and send them to a cloud-based platform for storage and analysis. A slightly different yet interesting idea was presented by Fugiglando et al. [25] who proposed a solution which gathers vehicular data from a data logger integrated in the car and then through a scoring algorithm produced the devised “Driver DNA”.

The motivation for this study was originated from the studies presented in the above paragraphs. The purpose is to propose an infrastructure capable to stream, store and analyze data in a reliable and fast way. To this end, the next Sections of the present study feature the description for the equipment and the infrastructure developed, the clustering procedure applied on the gathered data in order to label the dataset as well as the experimentation results of different ML and DL algorithms in order to identify which of them are more efficient and suitable for this specific application of driver behaviour classification. It is worth mentioning that the data used for this study is collected through actual operating vehicles which belong to the fleet of a major highway operator. The main idea is to provide an overview of a holistic solution for the driver classification application by presenting the in-vehicle equipment, the streaming process and finally the big data and cloud infrastructure developed and deployed so as to facilitate the collection, storage and analysis needs. Alongside the technical description of the proposed solution, a detailed presentation of how machine learning and deep learning algorithms perform for the classification of driving behaviour is also provided. The algorithms evaluated in the context of this study are well-known ML and DL algorithms. This study does not aim to provide a new algorithmic approach or a brand-new algorithm but a holistic approach of an integrated platform which gathers, stores, process and analyzes data coming directly from vehicles.

## 3. Data Collection and Analysis Infrastructure

### 3.1. In-Vehicle Equipment

The controller area network (CAN) was adopted by the automotive industry in 1993 and since then it is integrated in every vehicle manufactured so as to gather data from the vehicle’s sensors [26]. In 1993 CAN became an international standard known as ISO11898, later revised in 2015 [27]. Almost of all new vehicles are equipped with an OBD II standard interface which can host an OBD II decoder in order to collect, interpret and transmit data to other devices such as smartphones. For the purposes of this study and specifically the implementation of the proposed framework, the five different vehicles deployed for trials have an OBD II decoder installed which communicates via Bluetooth with a smartphone.

The OBD II decoder used integrates the microcontroller ELM-327 [28]. The ELM-327 is an OBD to RS232 interpreter which is commonly used in most commercially available OBD II decoders. The ELM-327 supports SAE J1850-PWM, SAE J1850-VPW, ISO 9141-2, ISO 14230-4 (slow), ISO 14230-4 (fast), ISO 15765-4 (CAN), SAE J2411 (SWCAN), SAE J1939 (250 kbps) and SAE J1939 (500 kbps) according to ELM electronics [29].

The OBD II decoder used for the purposes of this study it is the Konnwei KW-903 model which has been selected because of its small form factor as well as the support of connectivity via Bluetooth with a smartphone. The data that can be retrieved via the Konnwei KW-903 are shown in Table 1 [30].

**Figure 2:**
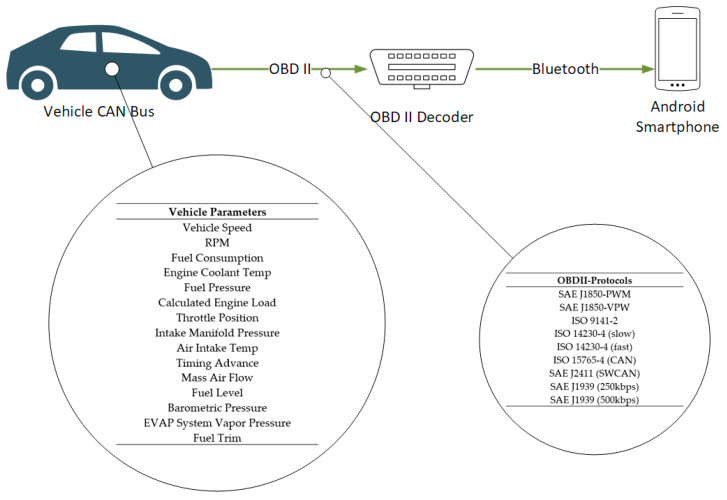
In-car equipment layout.

A selection of these parameters is retrieved and then transferred to the cloud-based hybrid data management platform. The selection of these features is described in detail in Section 4. The OBD II decoder described above transmits via Bluetooth the data to an Android 9.0 smartphone. The Android smartphone is connected to the Internet and relays the data to the cloud-based platform discussed in the next subsections. The data collection through the smartphone is carried out by utilizing an Android application with a typical user interface (UI) which the user/driver can use to check the connection status and the measurements in real-time. This application is just for monitoring reasons in order to ensure that the data transmission through the OBD to the smartphone and to the cloud-based platform is up and running. The data transmission to the cloud-based platform can be performed either automatically every five seconds or manually initiated by the driver. An abstract layout of the car equipment employed for the purposes of this study is shown in Figure 2.

### 3.2. Cloud-Based Data Management and Analysis Platform

The solution proposed in this study promotes a cloud-based platform which consists of two main components: (i) the streaming module and the (ii) the hybrid big data management and analysis module. These two modules operate together in order to provide an integrated solution for data streaming, storage and analysis.

#### 3.2.1. Data Streaming Module

The data streaming module is responsible for the continuous and uninterrupted data flow from the vehicle to the hybrid data management and analysis component and vice versa. This streaming module utilizes Apache Kafka, an open-source library for data processing and analyzing [31]. The three main functionalities of Apache Kafka are: (i) publication (write) and subscription (read) to streams of events; (ii) storing of streams of events and (iii) processing of streams of events. Kafka is designed to operate in a distributed way in order to be capable to handle vast data streams. Thus, Kafka provides high reliability and fault tolerance as well as scalability due to its distributed nature. The basic concepts/terms of Kafka are summarized below in Table 2.

**Figure 3:**
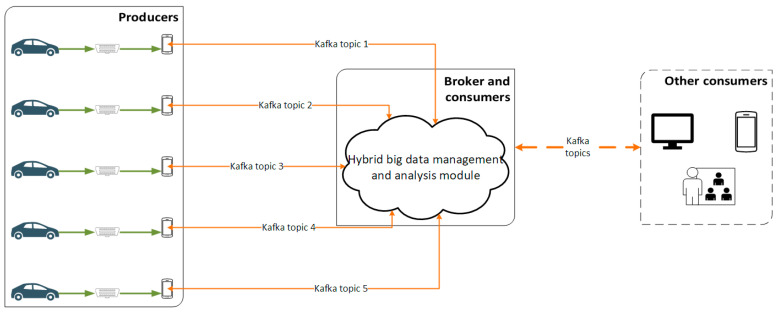
The Kafka streaming process implemented in the present study.

For the purposes of this study the role of producers is held by the five different vehicles equipped with data gathering and streaming equipment described in Section 3.2.1. Each of the vehicles establishes a different topic, which the consumers can subscribe to monitor. The broker is hosted in the cloud-based platform and regulates the data streams from the producers to consumers. Also, the consumers of the Kafka reside in the hybrid big data management and analysis module, which contains the relational and non-relational databases and the PySpark analysis component as described in Section 3.2.2. Moreover, the monitoring and operational systems of the administrator of the highway, the drivers through an application and whoever is authorized to access the data for monitoring and analysis purposes can be considered as the consumers of this data. The data flow of the streaming module is depicted in Figure 3.

#### 3.2.2. Hybrid Big Data Management and Analysis Module

The hybrid big data management and analysis module hosts both a relational and a non-relational database as well as an analytics engine as mentioned above. This module is designed in such a way so as to facilitate the primary requirements of a big data platform. These primary requirements and design principles followed for the development of this module are based on the main principles of the big data definition as stated by Andrew McAfee and Erik Brynjolfsson [32], namely Volume, Velocity, and Variety. More specifically, the big data management module collects and stores vast amounts of data, streaming from the five vehicles as described above and is, also, capable of hosting more vehicles or different data streams. Thus, the hybrid big data module is compliant with the Volume principle. As far as the Velocity principle is concerned, the module is designed to operate over distributed hardware resources in order to enhance, in terms of speed, its processing capabilities. Last but not least, the Variety principle mainly focuses on the ability of the module to handle various types of data, the format of which does not necessarily need to be standardized. In this context, the data coming from the vehicles are provided in several different formats as can be seen in Table 3. Moreover, the module is not limited to data streams coming from vehicles and used for the purposes of this study but also can collect, store and process any other data format coming from different streams.

In addition to the 3 Vs (volume, velocity, variety) described above, the hybrid big data module is designed and developed in such a way, so as to be highly reliable and future-proof. First of all, this module is designed to be high-reliable, able to operate smoothly without any external intervention. This means that the platform is capable to handle unexpected situations with least or ideally with no downtimes. This is achieved for example, by enabling the platform to recognize unexpected errors in software terms so as to auto-restart its services whilst at the same time the error is logged. This procedure provides useful information to the IT personnel in order to identify the cause and try to minimize the possibility of future occurrence. Also, this module can be distributed to several different hardware resources so as to increase its computational capabilities and its availability due to redundancy. Another characteristic of this module worth mentioning is its scalability capabilities which ensure that it can be easily expanded and lift any limitations that may arise when catering for increased storage and analysis needs while at the same time ensure that it can be adopted to different applications other than DBA.

The module under consideration is comprised of four basic widely used software components such as MySQL [33], MongoDB [34], Hadoop [35] and PySpark [36] which are described in what follows. Those components allow to acquire, store, and analyze data in a way that is agnostic to the sources from which data originate. In this sense, this very module can host and process data from different domains and not only vehicular data that are used for the purposes of this study. Last, but not least, this module is designed as a black box and can be hosted to any platform or framework requiring nothing else than just a “one-click” initialization

The MySQL is used as a relational database in order to provide near real-time analysis and data visualization. On the other hand, the MongoDB is selected so as to handle historical and batch analysis as well as management of the collected data. The development of this hybrid data management scheme is suitable for big data management, manipulation, and analysis operations. This approach, encompassing both relational and non-relational databases, enables us to harness both the benefits of big data such as storage capabilities and historical analysis for learning processes of the machine and deep learning algorithms as well as the fast response of the relational databases for instant visualization of live data and simpler statistical analysis.

**Figure 4:**
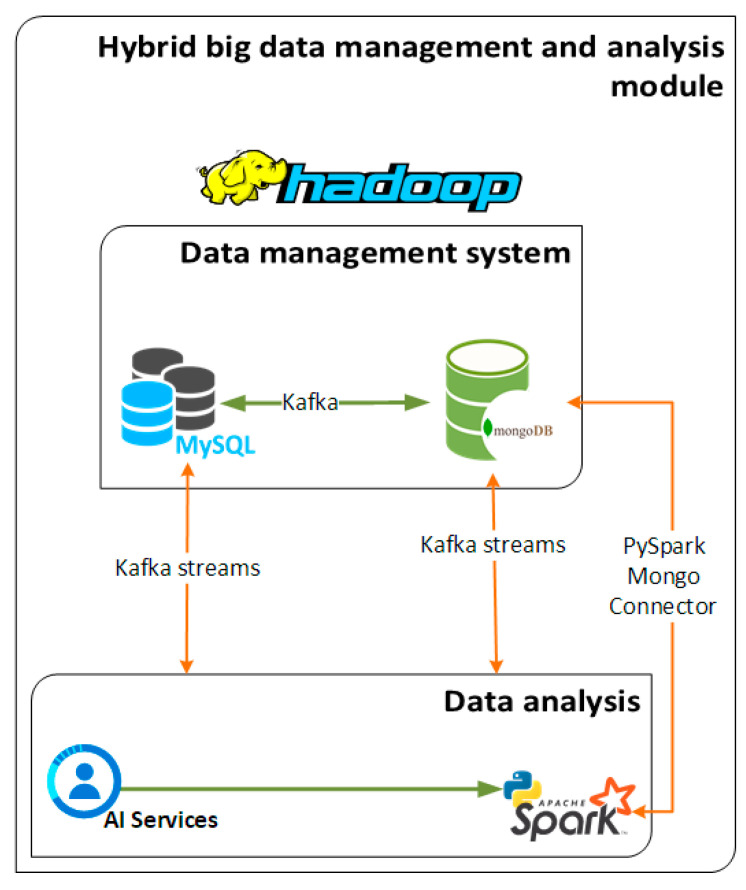
Hybrid big data management and analysis module.

Another basic component of the discussed module is the big data operational framework needed to serve both the MongoDB as well as the PySpark analysis module. Hadoop is the most well-known and widely used framework for big data operations and was the one selected for the purposes of the developed solution. The integration of the Hadoop Ecosystem in the hybrid big data management and analysis module offers an interface for the smooth operation of the NoSQL Database, described above, as well as for any big data operation required within the cloud-based platform. Together with the Hadoop framework the PySpark data analysis component is in charge of the analysis of the streamed and stored data. The PySpark module takes as input the machine and deep learning models for data processing purposes and then provides the outcomes of the analysis and the predictions back to the employed databases and to the corresponding Kafka topics so to be available to the consumers of each topic. The ML and DL algorithms implemented, used and tested for the purposes of this study as well as the acquired results are explained in more details in the next sections of this paper. A conceptual illustration of the hybrid management and analysis module is shown in Figure 4.

## 4. Dataset and Algorithms/Methods

### 4.1. Dataset Description and Data Clustering

OBD sensors are on-board computers (after 1996 every car or truck manufactured is legally required to have one installed) responsible for collecting and monitoring vehicle related data [37]. For the present research purposes, the data used for analysis were collected from five distinct vehicles, which belong to a major highway operator in Greece. The current dataset is composed of four million data instances collected within a specific time frame. After the application of preprocessing methods over it, any zero values are removed in order to isolate and analyze only the time periods within which each vehicle was not idle or stopped. As a result, the provided dataset consists entirely non-idle vehicle data. These data are collected and sent to the hybrid platform, described in Section 3, using a (defined) threshold for repetitive activity every five seconds. Moreover, till the time of writing this paper, the platform had already collected and stored over 20 million data points into the MongoDB where the dataset sample responsible for the ML models training and the analysis results was extracted after the preprocessing processes, as previously mentioned. Table 3 presents the features included in the dataset as was sent from the OBD decoders of the vehicles to the hybrid platform. Table 3 includes the feature name, the unit of measurement and the corresponding description.

Apache Spark consists one of most important modules integrated into the cloud-based platform, as was briefly described in Section 3, i.e., the module responsible for the implementation of the machine learning algorithms. The main reason of the integration of the current component is the configuration of a data feature extractor, in order to manage the large volume of data that the system is called to deal with, in the course of time. In fact, the integration of the data feature extractor is very important and is a desirable module for any large volume data management application. Also, from a mathematical point of view, the above provides maximization of the model’s ability to classify patterns by enabling scoring functions and thus improving the overall performance of the model. Feature extraction procedures are of primary importance for the reduction of the complexity of the input dataset, which significantly determines the success or failure of the current ML model in terms of accuracy and loss. As was previously mentioned, the current procedure is important for the implementation of the proposed machine learning algorithms in order to make patterns more visible, so as to enhance the efficiency of the applied algorithms. The feature selection process can be applied in both supervised and unsupervised methods in order to reduce the number of input variables. The main difference between these two methods lies on the selection of the features that are based on the target variable or not. For the purposes of this study, the supervised feature selection techniques to the target variable and more specifically filter-based feature selection which involves statistical-based feature selection methods were applied. Specifically, using statistical metrics, the correlation scores and/or the dependence between the raw input variables are filtered through the (feature) extractor component and the most relevant are eventually selected. The data unit that feature extractor is configured to handle is a multidimensional time series of 20 s if new data has been received by the platform consumer. PySpark was configured to check every 20 s if new data has been received by the platform consumer. As a result, irrelevant and redundant features, which can increase the computational cost and “disorient” the training model, can be removed. Thus, less computation resources are required and thus, the training dataset is smaller and dedicated to the training needs.

Table 4 lists the final dataset structure after the data feature extraction process is carried out, providing PySpark with the data volumes as streamed by the vehicles described in the Section 3.

After the preprocessing of the data through the PySpark module, an unsupervised clustering ML algorithm is applied to the current dataset, with the view to adopt a data-labelled approach, based on the rpm and speedOBD values. The selection of these two attributes from the data available was performed due to the fact that the vehicles deployed in this very study are equipped with fueled powered internal combustion engines (either gasoline or diesel). As it is widely known the fuel consumption of such engines is highly reliant to speed and rpm [38]. Often, high acceleration patterns lead to high rpm, a behaviour that is classified as aggressive driving style, resulting in higher fuel consumption and CO_2_ emissions and thus to a pollutive way of driving [39]. Nevertheless, it is worth mentioning that although the labelling is visualized using the rpm and speed values for reasons of comprehension and graphic overview, all of the values of the available dataset are taken into consideration, i.e., are provided as input to the clustering algorithm in order to label the data. From a technical point of view, clustering is an unsupervised machine learning algorithm, by which data instances are grouped in a way that similar observations are closer to each other and are able to identify clusters from a massive amount of data instances included in the provided dataset. Additionally, it is designated as an ‘unsupervised’ method since the training process is not implemented using labeled data. Instead the data are clustered based on the underlying patterns and properties. A variety of clustering algorithms, such as K-means, DBSCAN and hierarchical clustering can be found in the literature. For the current study, the K-means algorithm was selected, as (one of) the oldest and most approachable algorithms in terms of implementation. One of the most important key points for the implementation of the current algorithm involves the optimal number of clusters to be identified or “K”. A term (an artificial datapoint specifically, generated by the algorithm implementation), commonly known as “centroid” or the average of data points, provide a sense of the partition of the current data. In other words, the specific dataset will be split/partitioned into “K” clusters in a way that the distances between the centroids and instances will be minimized. After the preprocessing input from the Feature Extractor (PySpark) takes place, the next step is the determination of the optimal number of clusters. The so-called Elbow Method is executed in order to provide exactly this optimal number of clusters “K”, by minimizing the sum of squared errors [40,41]. The results of the clustering procedure are shown in detail in Section 5.

### 4.2. Machine and Deep Learning Algorithms for Data Analysis

Machine learning methods are traditionally applied into projects and datasets that involve the prediction of a target value, attribute or uncovering trends. In these types of applications, data is used to assist machines to be trained with new patterns that they can be later used to perform an accurate prediction on newly added input data, at distinct time instances [42,43]. The most popular machine learning algorithms include linear regression, decision trees, support vector machines (SVMs), naïve Bayes, discriminant analysis, neural networks and ensemble methods. The current study applies supervised machine learning methods in order to enable a training process using the provided, labelled training dataset. Specifically, the algorithms that are examined in terms of accuracy and loss of the provided model(s) include: logistic regression, SVM and random forest.

Lately, deep learning is gaining high popularity due to its ability to perform well, in terms of accuracy and precision, during training processes with huge amount of data. Deep learning algorithms are used for more complex datasets and architectures such as object, signal and/or image identification. Commonly used deep learning algorithms include recurrent neural networks (RNNs), multiple layer perceptron (MLPs) and reinforcement learning (deep Q networks).

Machine learning algorithms are faster when it comes to training new ML models and require less computational resources, hence result in quicker results in terms of execution time. In order to improve the model’s accuracy, it is important to apply feature preprocessing on the studied dataset in order to reduce its initial complexity and provide more visible patterns through the learning procedure.

On the other hand, deep learning algorithms require more time for execution as well as increased hardware and computing resources in comparison with ML algorithms. Deep learning algorithms are considered as a subset of the broader machine learning domain that are able to achieve higher-level feature learning in an incremental manner, decreasing in this way the need of extra effort for applying feature preprocessing methods [42,43]. DL techniques involve a hidden layer architecture, where each node (or hidden layer) provides a weight that symbolizes the correlation between the model’s output and the weight’s adjustment during the training process.

Providing a comparison statement between the aforementioned methods, deep learning achieves better results, in terms of accuracy and loss, in contrast with other traditional machine learning methods, in cases where the input data size increases over time, as the considered case where the OBD sensors from the vehicles continuously send data. Furthermore, the evolution of parallel computing methods with the integration of powerful GPUs that leads to high-end infrastructures, provides higher capacity and computational power, key features in order to achieve better training results, in terms of time [42,44], when deep learning techniques are applied. Another major issue that needs to be taken into account during the selection of machine or deep learning methods is the existence and the importance in the first case (machine learning methods) of a hardcore feature preprocessing in order to drop specific, useless features with a view to reducing the input dataset and eventually reduce the complexity of the procedure. On the other hand, deep learning methods eliminate the importance of feature extraction by detecting high-level features from the provided dataset introducing useful insights on the data [43], which is ignored through machine learning methods.

**Figure 5:**
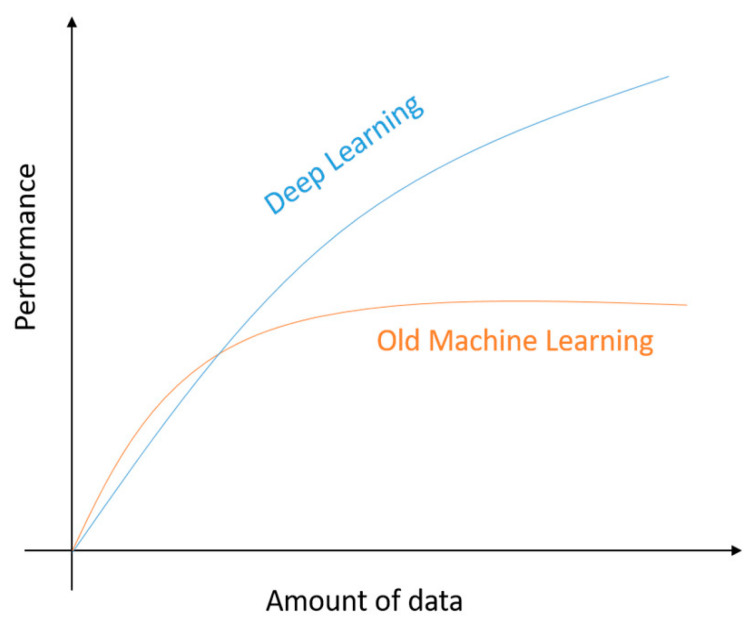
Performance of traditional machine learning methods and deep learning methods with respect to the amount of data [45].

In the context of the current study, three traditional machine learning algorithms were implemented and compared i.e., logistic regression, support vector machine (SVM) and random forest (RF) as well as two deep learning methods including two of the most popular ones, and more specifically the multiple layer perceptron (MLP) and the recurrent neural network (RNN) algorithms. The main reason for the variety of multiple algorithms implementation is to provide different benchmarking results for the current size of dataset, which is anticipated to increase during time, as the vehicles continue to send data back to the cloud-based platform. The rapid increase of the amount of data is expected to slow down the performance [45] of the traditional machine learning methods, whilst deep learning methods are not expected to be affected by the dataset size increase as shown in Figure 5. Consequently, the current study provides different comparison results for multiple models in cases where the amount of data is still under a specific threshold, where the suggested architecture can evaluate and select the most appropriate model, taking into account the actual dataset size and format. Also, to the best of our knowledge, there is not a direct comparative study for both machine learning and deep learning models regarding driver behaviour analysis as described by the authors of [45].

The aforementioned algorithms were benchmarked using the following metrics: (i) loss; (ii) validation loss; (iii) accuracy; (iv) validation accuracy; (v) F1 score; (vi) area under the ROC curve (AUC); (vii) precision; (viii) recall and (ix) execution time. The loss metric refers to both the training set (loss) and the validation set (validation loss) and is calculated as the sum of the distances of the predicted value from the real values. The accuracy metric is practically the number of correct predictions divided by the total number of predictions performed as shown in Equation (1) [46].

The accuracy metric was applied for both the training and the validation set. In addition to the loss and accuracy metrics, the F1-score is calculated through the precision and recall values. Precision and recall metrics indicate the rate of true positives in the predicted positives and actual positives respectively as shown in Equations (2) and (3). Thus, the F1-score is the harmonic mean among precision and recall and is calculated as shown in Equation (4) [46]. The next metric i.e., the area under ROC curve (AUC), as indicated by its name, utilizes the receiver operating Characteristics (ROC) curve. ROC graphs are two-dimensional graphs, that feature true positive (TP) rate (Equation (5)) on their *y*-axis and false positive (FP) rate (Equation (6)) on their *x*-axis. Every ROC graph depicts the tradeoff between the benefit and cost (TP vs. FP). In order to compare different methods using the ROC curve the metric usually used is the AUC as mentioned by Hanley et al. in [47]. The value of the AUC lies between 0 and 1 because it is actually a portion area into a unit square. The higher the value of the AUC the better the classifier is. Considering the solution proposed in this study, AUC is a metric which can provide an evaluation of the methods used to classify a data point as an eco-friendly driver or not. Last but not least, alongside the evaluation metrics, the time required for the execution of each algorithm is presented in order to provide an insight on the computational cost and highlight the difference of ML and DL algorithms in this term. All the results are collected and presented in Section 5 in more details:

(1) $$Accuracy=\frac{TP+TN}{Total\;number\;of\;samples}$$

(2) $$Precision=\frac{TP}{TP+FP}$$

(3) $$Recall=\frac{TP}{TP+FN}$$

(4) $$F1\text{-}score=2\times \frac{1}{\frac{1}{precision}+\frac{1}{recall}}$$

(5) $$TP\_rate=\frac{positives\;correctly\;classified}{total\;positives}\;$$

(6) $$FP\_rate=\frac{negatives\;incorrectly\;classified}{total\;negatives}$$

## 5. Results

The algorithms were developed and implemented in Python using the scikit-learn framework [48] for the machine learning algorithms and the tensorflow framework [49] for the deep learning algorithms. The testbed used for trials was an Arch Linux OS system with i9-9900k CPU, 2080ti GPU and 32 GB of RAM, equipped with a 500 GB SSD drive. The current architecture can be expanded in the future, across multiple computer clusters, where the actual hardware will consist of the master node and worker and client nodes to analyze and store large volumes of data accumulated by the continuous streams from car sensors. The evaluation was conducted for both ML and DL algorithms in a preprocessed and labelled dataset. The labelling procedure was performed through unsupervised clustering as mentioned in further details in Section 4. The clustering results are presented and explained in detail in the next subsection. Following the clustering results and the labelling procedure of the given dataset, the results of the evaluation of the several ML and DL algorithms are provided.

### 5.1. Labelling of the Dataset via Clustering

**Figure 6:**
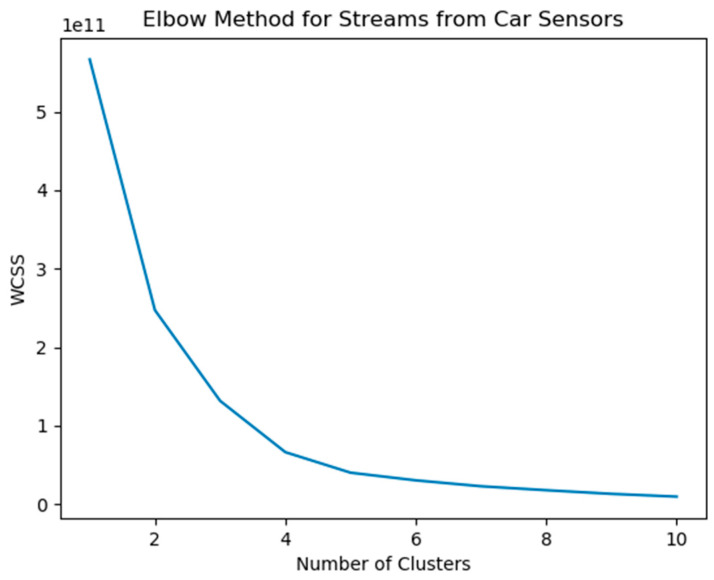
Elbow method implementation for streams from car sensors.

Figure 6 depicts the results of the current elbow method implementation, over the studied dataset. The optimal number of clusters “K” is equal to K = 2, for the current RPM-SpeedObd values combination.

**Figure 7:**
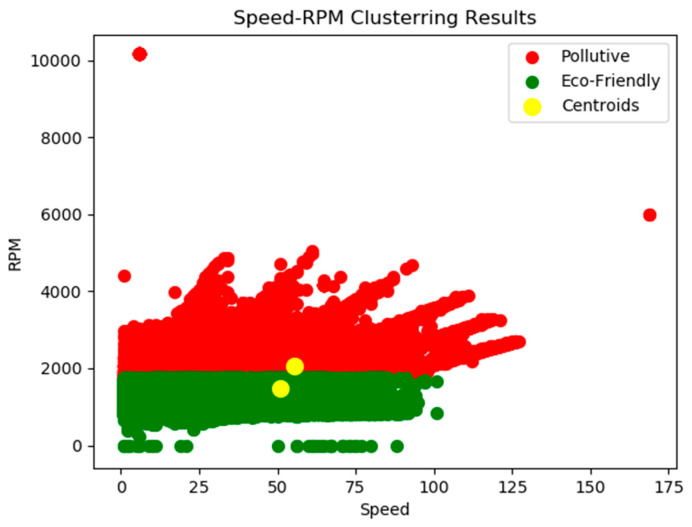
Clustering algorithm implementation for streams from car sensors.

Therefore, the K-means algorithm, following the fitting process of the model, will visualize two distinct clusters in a two-dimensional plot, which will be used for further analysis purposes of the present study. The results of the clustering implementation are depicted in Figure 7.

As was previously described there are two distinct classes: the green one can be characterized as the one including eco-friendly drivers while the red one consists of drivers with driving behaviour that can be characterized as aggressive or pollutive. This labelling process provides the capability to apply supervised ML and DL algorithms and evaluate their performance in the given dataset.

### 5.2. Machine and Deep Learning Algorithms Evaluation Results

After the labelling procedure has been performed and the clustering results presented previously have been acquired, the new labelled dataset was provided as input to the ML and DL algorithms mentioned in Section 4. Table 5 provides a brief description of the applied (ML and DL) algorithms in addition to the hyperparameters that were selected for each distinct model as a result of the fine tuning/hyperparameter optimization procedure in order to explore the possibility of achieving enhanced forecasting accuracy with reduced losses.

Table 6 contains the values for each of the eight (8) metrics used, as these are mentioned in Section 4, for each of the three (3) ML and two (2) DL algorithms engaged for the needs of this study. It is worth mentioning that regarding the loss and accuracy metric the calculation was carried out for both the training and validation set whilst for the rest of the metrics (F1 score, AUC, precision, recall) this was performed for the validation set only. More specifically, the training set consisted of the 2/3 (67%) of the whole dataset (approximately 2.67 million records) while the validation set consisted of the remaining 1/3 (33%) of the whole dataset (approximately 1.33 million records).

**Figure 8:**
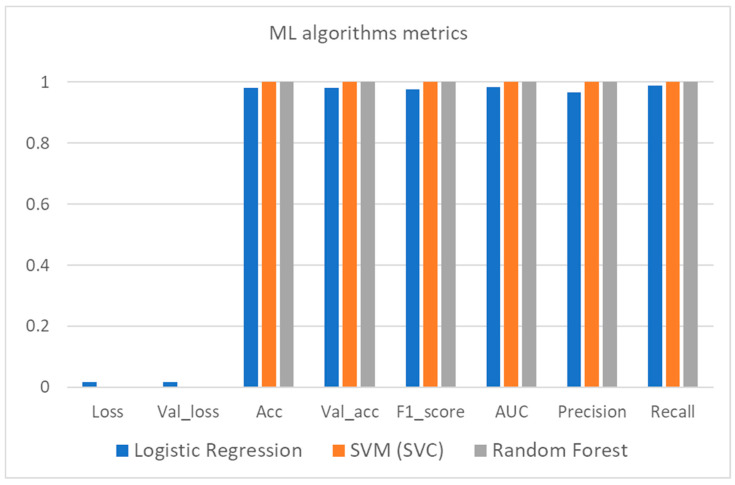
ML algorithms metrics bar plot.

**Figure 9:**
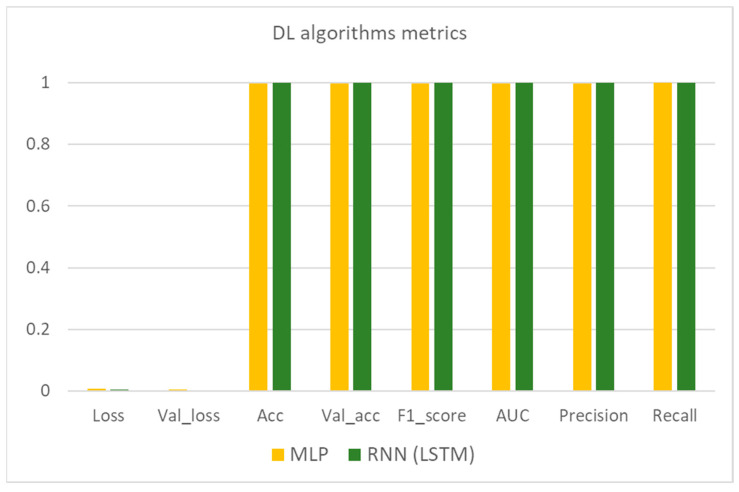
DL algorithms metrics bar plot.

**Figure 10:**
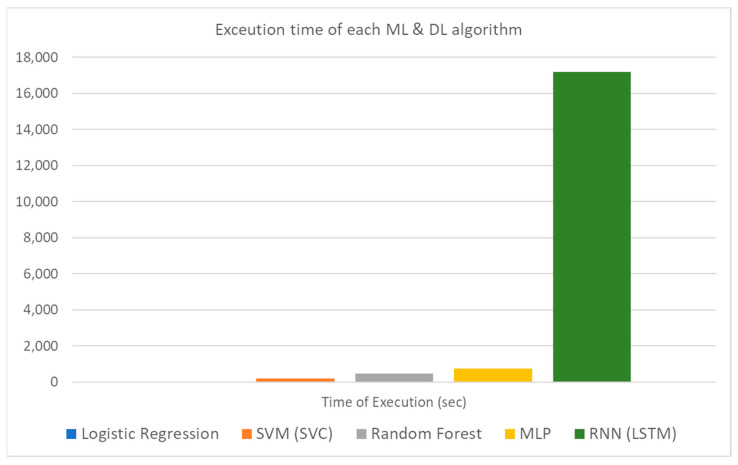
ML and DL algorithms execution time bar plot.

The results in Table 6 indicate that all of the benchmarked algorithms feature remarkable results in the prelabelled dataset and thus can be used for real-time evaluation of new data coming from vehicles. It is worth mentioning that in the ML algorithms family, SVC and random forest performed slightly better than logistic regression in terms of performance metrics but at a higher computational cost. As far as the DL algorithms are concerned, the LSTM is also slightly better, but the execution time is quite high compared both to MLP as well as to the aforementioned ML algorithms. It is worth underlining that the loss and validation loss values for both ML and DL algorithms are very similar for each algorithm considered and this consists a useful indication that there is not any overfit or underfit phenomenon during the training process. A graphic illustration of these results is depicted in Figure 8, Figure 9 and Figure 10.

As can be seen by these three figures and as already mentioned, the performance metrics of the studied algorithms are quite encouraging. The biggest difference is spotted in the execution time where the LSTM algorithm features a significantly increased time. This result is expected due to the complexity of this specific method. The reason that both ML and DL techniques were evaluated in this study lies on the nature of the input data. Traditional ML algorithms perform quite well even in a single hardware unit but considering that the pace of the incoming data is quite rapid, the size of the accumulated dataset as well as the rapid changes underpin the use of distributed methods and hardware resources so as to achieve the desired efficiency of the final selection. Thus, in this light, the experimentation on DL algorithms so as to expand and scale up the initial idea into a new generation, future proof, framework is mandatory.

Several interesting studies with worth-to-mention results can be found in this domain, as already mentioned in Section 2. Chen et al. [10] combined PCA and multiple linear regression and their method featured an accuracy of around 97%. Also, authors in [17] used the Strategic Highway Research Program 2 (SHRP2) naturalistic driving study (NDS) dataset and compared several different algorithms in order to conclude that the Random Forest classifier with a 10-fold cross validation achieved average accuracy of around 90% and average F1-score of 0,945. Also, Abdelrahman et al. [16] used the same dataset as the aforementioned study (SHRP2 NDS) and the RF classifier they proposed achieved 93.2% accuracy, 95.08% precision and 93.5% recall. Moreover, in [18], Chen et al. followed a comparative analysis of different machine learning algorithms in data acquired through an OBD II decoder in a single car. Their results indicated that the top three ML algorithms were RF, decision tree and gradient boosting with validation (test) accuracy of above 95% [18]. To the best of our knowledge, despite the many research efforts on driver behaviour analysis using machine learning algorithms, there are no similar comparative studies of both ML and DL algorithms in this specific domain. Table 6 includes a direct benchmarking between different machine and deep learning algorithms for the same dataset used in this study and their respective performance.

## 6. Conclusions and Future Work

The aim of this study was to research and present a proof-of-concept holistic approach for driver behaviour analysis based on vast streams of vehicular data by testing and evaluating different known machine and deep learning methods. To this end, various machine and deep learning algorithms were presented, implemented, applied, and evaluated in order to study their performance on vast and rapid streams of vehicular data. The results underpinned that the engagement of both of ML and DL methods is suitable for the given dataset. The metrics used are the loss, accuracy, the F1-score, precision, recall, the AUC and the execution time. The main differentiation between all the known tested methods was found to reside in the execution time between the ML and DL algorithms, as was anticipated. This occurs due to the fact that both ML and DL algorithms are executed on the same hardware as described in Section 5. This cannot be considered as a drawback neither for the purposes of the solution proposed in this study nor for the DL methods because as mentioned the deployed cloud-based framework is designed with scalable and distribution capabilities.

The overall idea of this very study was to present and examine the design and the functionality of an actual real-life deployed platform with real data streams coming from vehicles which are running on shifts and belong to a major highway maintenance and operation administrator in Greece. The state-of-the-art tools engaged for the needs of this research study were selected based on the recent literature review conducted in Section 2, with the aim to ensure that the proposed solution is future-proof and technologically open. To this end, the study does not aim at providing a new algorithm for driver behaviour analysis but rather an integrated platform which combines state-of-the-art technologies such as streaming processes, big data infrastructure as well as ML and DL modules for data analysis.

Despite the fact that the autonomous vehicles gain more and more ground day by day, driver behaviour analysis is still an active and open research domain. Thus, the outcomes of this study could be expanded in the future by applying training results of the examined ML and DL algorithms for real-time classification of the drivers, according to their driving behaviour. Based on clustering analysis described previously, a future approach could also involve the execution of the actual unsupervised analysis over different pairs of values of the specific dataset (except of the already provided pair of rpm-SpeedOBD), in order to provide additional labels/profiles in the same logic. A meta-profile engine could be a (future) result/proposal of the algorithmic combination of the provided labels from the current dataset, where the evaluation of each driver could be returned in real time through the suggested cloud-based platform, again, based on continuous streams from car sensors, as were described in previous sections. Also, further data and tools, such as semantic technologies and complex event processing (CEP), could be engaged in order to gain more useful insights not only on the driving behaviour but also the ongoing road and traffic conditions. In this light, data coming from the highway itself or by satellites, such as traffic density, weather forecast etc. can be given as input through different streams and after proper analysis can reveal hidden patterns and information which could eventually provide useful tips and insights in order to render the drivers and the authorities aware of dangerous and risky situations. A more holistic approach of such tools and technologies could lead to significantly increased safety in highways as well as to optimized performance in environmental and traffic terms. On the other hand, simultaneously with the exploration of new advanced approaches, research on the drawbacks, risks and dangers that come along with the evolution of transportation systems should also be conducted. Thus, researchers should also focus on the computational efficiency of those methods, on trustworthiness of the decision-making AI as well as on cybersecurity aspects and measures.

## Figures and Tables

**Table 1:** Konnwei KW-903 parameters reading in default settings.

Vehicle Parameters
Vehicle Speed
RPM
Fuel Consumption
Engine Coolant Temp
Fuel Pressure
Calculated Engine Load
Throttle Position
Intake Manifold Pressure
Air Intake Temp
Timing Advance
Mass Air Flow
Fuel Level
Barometric Pressure
EVAP System Vapor Pressure
Fuel Trim

**Table 2:** Kafka terms and description.

Kafka Term	Description
Topic	A group of data flows of a specific category
Producer	Applications which publish messages in one or more Kafka topics
Broker	Applications which receive and transmit a message from a producer to a consumer
Consumer	Applications which subscribe (read) the messages from one or more topics through the brokers

**Table 3:** Data as streamed by vehicles through the smartphones.

Feature Name	Unit of Measurement	Feature Description
timestamp	date & time	the timestamp of the measurement
lat	degrees	the latitude of the vehicle’s position
lon	degrees	the longitude of the vehicle’s position
altitude	meters	the altitude compared to sea level
accuracy	number	the accuracy of the vehicle’s geolocation
bearing	degrees	the direction of the vehicle
speedGPS	km/h	the speed of the vehicle according to the GPS
speedOBD	km/h	the speed of the vehicle
vinNumber	number	the unique vehicle’s identifier
rpm	rounds/min	the engine’ rounds per minute
relThrottle	percentage	the throttle’s position percentage
intakeTemp	°C	the environmental temperature
engineTemp	°C	the engine temperature
fuelType	list	the type of the fuel used by the vehicle
fuelLevel	percentage	the level of the fuel in the vehicle’s tank
engineRuntime	hours	the hours since the engine started
pendingTrouble	binary	declares if there is an emergency situation
carPlate	string of characters	the license plate of the car

**Table 4:** Final dataset structure after feature extraction procedure using PySpark.

Variable	Unit	Type
altitude	meters	integer
accuracy	number	double
bearing	degrees	double
speedGPS	km/h	integer
speedOBD	km/h	integer
rpm	rounds/min	integer
relThrottle	percentage	integer
intakeTemp	°C	integer
engineTemp	°C	integer
fuelType	list	integer
fuelLevel	percentage	integer
engineRuntime	hours	integer

**Table 5:** Examined ML and DL algorithms and their parameters.

Type	Algorithm Name	Parameters
Machine Learning	Logistic Regression	C: 1.0, penalty: ‘elasticnet’, tolerance: ‘False’, fit_intercept:‘1e-4’, class_weight: ‘balanced’, solver: ‘liblinear’, multi_class: ‘ovr’
	SVM (SVC)	C: 1.0, kernel: ‘linear’, degree:1, gamma: ‘scale’, class_weight: ‘balanced’, decision_function_shape: ‘ovr’
	Random Forest	n_estimators: 100, criterion: ‘entropy’, max_features: ‘auto’, class_weight: ‘balanced’
Deep Learning	MLP	hidden_layers: 4, optimizer: ‘adam’, loss: ‘binary_crossentropy’, epochs: 100, batch_size: 256
	RNN (LSTM)	hidden_layers: 2, units: 100, optimizer: ‘adam’, loss: ‘binary_crossentropy’, epochs: 100, batch_size: 512

**Table 6:** Result metrics of the tested ML and DL algorithms on the same dataset.

Type	Algorithm	Training Loss	Validation Loss	Training Accuracy	Validation Accuracy	F1 Score	AUC	Precision	Recall	Execution Time (s)
Machine Learning	Logistic Regression	0.018	0.018	0.982	0.982	0.977	0.984	0.966	0.989	39.706
	SVM (SVC)	0.000	0.000	1.000	1.000	1.000	1.000	1.000	1.000	211.080
	Random Forest	0.000	0.000	1.000	1.000	1.000	1.000	1.000	1.000	471.997
Deep Learning	MLP	0.006	0.005	0.997	0.998	0.998	0.998	0.998	0.999	758.756
	RNN (LSTM)	0.004	0.002	0.999	1.000	1.000	1.000	1.000	0.999	17,184.356

## Data Availability

Not applicable.
